# Induced Abortion and Associated Factors in Health Facilities of Guraghe Zone, Southern Ethiopia

**DOI:** 10.1155/2014/295732

**Published:** 2014-03-30

**Authors:** Gezahegn Tesfaye, Mitiku Teshome Hambisa, Agumasie Semahegn

**Affiliations:** ^1^Department of Public Health, College of Health and Medical Sciences, Haramaya University, P.O. Box 235, Harar, Ethiopia; ^2^School of Nursing and Midwifery, College of Health and Medical Sciences, Haramaya University, P.O. Box 235, Harar, Ethiopia

## Abstract

Unsafe abortion is one of the major medical and public health problems in developing countries including Ethiopia. However, there is a lack of up-to-date and reliable information on induced abortion distribution and its determinant factors in the country. This study was intended to assess induced abortion and associated factors in health facilities of Guraghe zone, Southern Ethiopia. Institution based cross-sectional study was conducted in eight health facilities in Guraghe zone. Client exit interview was conducted on 400 patients using a structured questionnaire. Bivariate and multivariate logistic regression analysis was performed to identify factors associated with induced abortion. Out of 400 women, 75.5% responded that the current pregnancy that ended in abortion is unwanted. However, only 12.3% of the respondents have admitted interference to the current pregnancy. Having more than four pregnancies (AOR = 4.28, CI: (1.24–14.71)), age of 30–34 years (AOR = 0.15, CI: (0.04–0.55)), primary education (AOR = 0.26, CI: (0.13–0.88)), and wanted pregnancy (AOR = 0.44, CI: (0.14–0.65)) were found to have association with induced abortion. The study revealed high level of induced abortion which is underpinned by high magnitude of unwanted pregnancy. There is requirement for widespread expansion of increased access to high quality family planning service and post-abortion care.

## 1. Introduction 

World Health Organization (WHO) defines unsafe abortion as a procedure for terminating unwanted pregnancy either by people lacking the necessary skills or in an environment lacking minimal medical standards or both [1]. According to WHO, worldwide unsafe abortions were estimated to be 21.6 million, almost all in developing countries. Each year, throughout the world, approximately 210 million women became pregnant and around one in 10 pregnancies ends in an unsafe abortion. The estimated annual number of unsafe abortions in Sub-Saharan Africa is 5.5 million. Although unsafe abortions are preventable, they continue to pose undue risks to a woman's health [2].

Millennium Development Goal (MDG) 5, announced in 2001, is an internationally agreed-upon imperative to reduce maternal mortality by 75% from its 1990 level by the year 2015. As a significant proportion of mortality is due to unsafe abortion, this goal probably cannot be met without specific and direct programmatic efforts to reduce the impact of unsafe abortion [3].

Unsafe abortion accounts for 13% of maternal deaths worldwide and as much as 25% in some countries [4]. Over 40% of the total deaths due to unsafe abortion have occurred in Africa making it the leading cause of maternal mortality in the region [5]. The maternal mortality ratio (MMR) in Ethiopia was estimated at 676 deaths per 100,000 live births in the year 2011 [6]. It is estimated that there are 3.27 million pregnancies in Ethiopia every year, of which approximately 500,000 end in either spontaneous or induced abortion [7].

In 2005, Ethiopia expanded its abortion law, which had previously allowed the procedure only to save the life of a woman or protect her physical health. Currently abortion is legal in Ethiopia under certain preconditions that include cases of rape, incest or fetal impairment, if the pregnancy endangers her or her child's life, or if continuing the pregnancy or giving birth endangers her life. A woman may also terminate a pregnancy if she is unable to bring up the child, owing to her status as a minor or to a physical or mental infirmity. Despite the implementation of the new law, almost six in ten abortions in Ethiopia are unsafe [8].

Reasons for resorting to unsafe abortion after unwanted or unplanned pregnancy were well established and reflected the status of women in the society. Some of the reasons are inability to support self and family, having already enough children, being very young, and being in school [9–11]. Lack of access to adequate family planning services is a major contributor to the global problem of unsafe abortion. A woman seeking treatment for incomplete abortion already may have experienced an unwanted pregnancy either as a result of not using contraception or method failure [12].

A nationally representative survey conducted in Ethiopia in 2008 revealed that an estimated 382,000 induced abortions were performed and 52,600 women were treated for complications of abortion. The annual abortion rate was 23 per 1,000 women aged 15–44 years and the abortion ratio was 13 per 100 live births, about 42% of pregnancies were unintended, and the unintended pregnancy rate was 101 per 1,000 women [13]. Another hospital based study in Ethiopia revealed 50% unwanted pregnancies and 25.6% induced abortion. Fifty-eight percent of the women who induced abortion terminated the current pregnancy either by seeking the help of untrained personnel or by themselves with no assistance [14]. A survey conducted in Harar, Eastern Ethiopia, showed 33.3% unintended pregnancies while induced abortion was found to be 14.4% [15].

Unsafe abortion is one of the greatest neglected problems of health care in developing countries and is a major medical and public health problem in Ethiopia [6]. Nevertheless, lack of up-to-date and reliable information on induced abortion distribution and its determinant factors has partly hindered the progress towards alleviating the problem in the country. Thus, the aim of this study was to assess induced abortion and associated factors in health facilities of Guraghe zone, southern Ethiopia.

## 2. Methods and Materials 

### 2.1. Study Area and Period

The study was conducted in health facilities of Guraghe zone, southern Ethiopia. Guraghe zone is one of the 13 zones in the Southern Nations, Nationalities, and Peoples Region (SNNPR). Its capital Welkite is located 156 km south west of Addis Ababa along the road from Addis Ababa to Jimma. The zone is divided into 13 administrative woredas with an estimated total population of 1.7 million. According to the zonal health bureau, the zone has 26 health centers, one zonal public hospital, two nongovernmental hospitals, and one Family Guidance Association (FGA) clinic. Eleven health facilities have already initiated post-abortion care services (two hospitals and nine health centers) in the zone. Eight health facilities (two hospitals and six heath centers) were included in the study. The study was conducted from January to March, 2010.

### 2.2. Study Design

The study employed facility-based cross-sectional study design. Facility based study was preferred because health facility is the most logical, cost-effective, and convenient place to conduct research on unsafe abortion where women with complications of unsafe abortion are treated despite having its own limitations [16].

### 2.3. Source Population

All women who received post-abortion care service in health facilities of Guraghe zone were included.

### 2.4. Study Subjects

#### 2.4.1. Inclusion Criteria

All women who received post-abortion care during the study period in the 8 selected health facilities were included in the study.

#### 2.4.2. Exclusion Criteria

Those patients who were unable to hear, mentally disabled, seriously sick, and were not volunteer to participate and patients whose gestational age was greater than 28 weeks were excluded from the study.

### 2.5. Sampling

#### 2.5.1. Sample Size Determination

The sample size was calculated based on the following assumptions: proportion of patients who seek post-abortion care (*P* = 50%) taken to get the maximum sample size, *Z* = 1.96 at 95% confidence interval, *d* = the level of precision (0.05), and non-response rate = 10%; then the total sample size for this study became 422.

#### 2.5.2. Sampling Procedure

All consecutively discharged cases from the selected health facilities during the study period were included in the study until the required sample was obtained for each heath facility as determined based on their patient load as recommended by WHO [16].

### 2.6. Data Collection Method and Tool

Interviewer administered structured questionnaire was used to collect the data. The questionnaire was developed after extensive review of similar studies done previously and adapted to the purpose of the study. The types of questions asked include “sociodemographic data,” “reproductive health history,” “reproductive intentions,” and “fertility control.” The questions were translated into local language and administered by the interviewers in a careful way so as to keep the patient at ease and in a manner showing respect to their opinion in a nonjudgmental approach. Information about induced abortion was elicited from the study participants by using the question “is the current abortion after an attempt to terminate the pregnancy?” and asking them to explain about “why (reason) they resort to induced abortion,” “the condition under which the induced abortion was performed,” and so forth. The data collectors were female diploma nurses. The clients were interviewed after discharge was decided and just before the client left the respective health institution as exit interview.

### 2.7. Study Variables

*Dependent Variable*. Induced abortion which refers to “post-abortion patients who admitted interference to terminate their pregnancy intentionally either on their own or by another person.”

*Independent Variables. *Independent variables include the following:

- sociodemographic variables;
- reproductive history (current pregnancy (wanted versus unwanted), previous abortions, and number of pregnancies).

### 2.8. Data Entry and Analysis

After collection of the data the responses were coded and entered into EPI Info version 3.5.1. Data were cleaned and then exported to SPSS version 15 for windows for analysis. Then, the frequency distribution of dependent and independent variables was worked out. A crude and adjusted odds ratio from bivariate and multivariate analyses was used to measure association between dependent and independent variables. Those variables which were found to be significant in the bivariate analysis at *P* value < 0.05 were taken to logistic regression model for further multivariate analysis. Then, logistic regression analysis was done to control confounding variables and to identify independent factors associated with induced abortion. Participants were allocated to cases (those having induced abortion) based on their response, so those who have admitted that the abortion is because of interference were allocated to “cases” and those who deny it as “noncases” in the logistic regression analysis.

### 2.9. Data Quality Assurance

The English version questionnaire was translated into local language Amharic and again back to English so as to ensure its consistency. The translated Amharic version was pretested in a health facility other than the selected facilities in 5% of the sample size that were not part of the main study. Modification was made on omitted, an unanswerable, or unclear questions accordingly. Supervisors daily check completeness, clarity, and consistency of the questionnaire. The data collectors and supervisors were trained by the principal investigator on the objectives of the study and how to make the interview, fill the questionnaire, and handle questions asked by clients during interviewing for three days.

## 3. Results

### 3.1. Sociodemographic Characteristics

A total of 422 patients were identified and 400 women participated in the study making the response rate 95%. Majority, 193 (48.25%), of the respondents were aged between 20 and 29 years. The mean age of the respondents was 25.3 years (SD ± 6.4). About half, 227 (56.8%), of the participants were married, 130 (32.5%) were illiterate, and 185 (46.3%) were housewives (Table 1).

### 3.2. Reproductive History, Family Planning Knowledge, and Practice

About 207 (51.8) of the respondents have history of one or two pregnancies including the current pregnancy that ended in abortion. The majority, 185 (46.5%), of the patients have no history of delivery. Sixty-eight (17%) of the respondents had previous history of abortion, which was experienced once in 86.8%, twice in 11.7%, and three times in 1.5% of the respondents. The majority, 302 (75.5%), of the post-abortion patients revealed that the current pregnancy which ended in abortion was unwanted while the remaining ninety-eight (24.5%) of the patients said it is wanted. Forty-nine (12.3%) of the patients admitted interference with the current pregnancy (Table 2).

**Figure 1:**
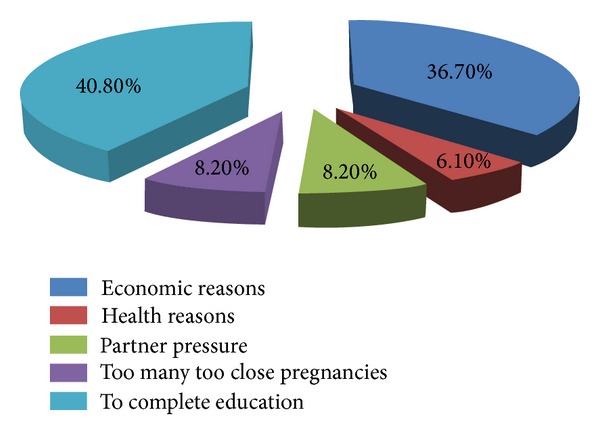
Reasons for interference with the current pregnancy among post-abortion clients in Guraghe zone, south Ethiopia, from January to March, 2010.

The majority of those who reported their last pregnancy as unwanted stated partner pressure and negligence to take contraceptives regularly as main reasons for the pregnancy. Among those who admitted interference to the current pregnancy reported place of interference as health institutions, patient's home, and inducer's house, 25 (51%), 22 (45%), and 2 (4%), respectively. Materials used to induce the pregnancy were herbs, 18 (36.7%); plastics, 15 (30.6%); and different medications in 11 (22.4%) of the patients. Twenty (40.8%) reported that interference was done by themselves followed by health workers, 25 (51%) (Table 3). Main reasons given for resorting to induced abortion by those who admitted interference were to complete education, 20 (40.8%), and for economic reasons, 18 (36.7%) (Figure 1).

Two hundred eighty-two (70.5%) of the study participants knew at least one family planning method, the most commonly known being oral contraceptive pills. Two hundred forty three (60.8%) of the participants have used contraceptive at least once in their life time. When seen by type of abortion, 33 (67.3%) of those who admitted interference and 210 (69.5%) of those with spontaneous abortion reported use of contraceptive at least once in their life time.

### 3.3. Fertility Awareness, Pregnancy Intention, and Receiving Family Planning Method

Majority of the clients, 123 (30.8%), stated that fertility will return within one month, 162 (40.5%) never want to be pregnant in the future, only 226 (56.5%) got post-abortion family planning method, and the most common reason for not getting family planning method was, 96 (24%), refusal and, 47 (11.8%), not counseled family planning (Table 4).

### 3.4. Factors Associated with Induced Abortion

In the bivariate analysis, educational status and number of pregnancies are associated with induced abortion. In multivariate analysis, those patients who have greater than four pregnancies were more likely to have induced abortion than those who have less than four pregnancies (AOR = 4.28, CI = (1.24–14.71)) and those patients who reported that they want the current pregnancy were less likely to have induced abortion (AOR = 0.44, CI: (0.14–0.65)) than those who do not want their current pregnancy. In addition, those patients who were in primary school and age group 30–34 were less likely to have induced abortion (AOR = 0.26, CI: (0.13–0.88)) and (AOR = 0.15, CI: (0.04–0.55)) (Table 5).

## 4. Discussion

In this study the magnitude of induced abortion was 12.3%. The main reasons for induced abortion were to complete education, 40.8%, and economic reason, 36.7%. More than three-fourth, 75.5%, of the current pregnancies were unwanted. Partner pressure and forgetting to take contraceptives were the main reasons mentioned for unwanted pregnancy. About 40.5% of the clients never want to be pregnant at all. More than half, 51%, of the induced abortions took place in health institution by health workers. Age 30–34 years, primary education, number of pregnancies, and wanted pregnancy had association with induced abortion.

Even though, 75.5% reported current pregnancy was unwanted in this study; only 12.3% admitted that the pregnancy was interfered. According to WHO, distinguishing between spontaneous and induced abortion among women who received care in health facility is difficult. Since women fear retribution or inadequate care, they are likely to deny unsafe procedures even in the face of the most obvious evidence. Therefore, WHO classified those women who admitted the pregnancy interference in the certainly induced abortion category and those women who states that the pregnancy was unwanted but denied interference in the category of possibly induced abortion [16]. As a result the magnitude of induced abortion may be underestimated. In general this is a sensitive area that respondents do not want to disclose. Due to this fact results from different studies show varying number of proportions between spontaneous and induced abortions. A study conducted in Isfahan, central Iran, revealed a similar result; 12% of the patients reported induced abortion [17]. Another study conducted in northwest Ethiopia revealed 4.8% prevalence rate of induced abortion which is much lower than our study [18], implying that induced abortion is a hidden public health problem affecting the reproductive health outcome status of women.

The current study finding shows partner pressure and negligence to take contraceptives as a main cause for the unwanted pregnancies which is similar to other studies [11, 19] in that contraceptive misuse or poor knowledge are the main cause for unwanted pregnancies. These facts show how the low socio-economic status of women in the community affects their decision making that risks their life temporarily or permanently. Women still lack the autonomy to make decisions about their pregnancy intention. As is the case with many other areas of reproductive health, husbands appear to be the primary decision-makers with regard to pregnancy. Furthermore, the main reasons for resorting to induced abortion in this study (economic reason and to complete education) were not different from the work of previous investigators [11, 20, 21].

In our study, 40.5% of the clients never want to be pregnant which is higher than study conducted in Addis Ababa where only 31.7% never want to become pregnant at all [19]. The number of women who want to limit pregnancy is still high in both studies which imply that there is a high family planning demand to space or limit their next pregnancy calling that post-abortion setting continues to be an important opportunity to provide post-abortion family planning counseling and initiate family planning methods.

The fact that 51% of the induced abortion was undertaken in health institutions by health workers might be due to the flareup of private clinics which leads to management in substandard settings and poor professional competence of health workers. According to a study in Mekelle, northern Ethiopia [21], health professionals undertook the inductions in 82.7% which is even much higher than this study. A study in Dare Selaam showed that most (79%) abortions had been induced at clinic or hospital (43.6%) by health care workers [22].

This study showed that being in primary school, age 30–34 years, having more than 4 children, and wanted pregnancy had association with induced abortion. Being in primary education is protective of induced abortion in this study. A study in north west Ethiopia showed a different result in that as the level of education increased there was an increase in the number of mothers who had induced abortion, particularly those who had a high school education were highly exposed to induced abortion [18]. This may be due to the fact that children in primary school were living with their family under strict parental supervision which prevents them from unwanted pregnancy which ends up with induced abortion.

Age 30–34 years [AOR = 0.15, (CI 0.04–0.55)] was negatively associated with induced abortion. A study done in Ghana [23] also showed the same result in which women with induced abortions were significantly within younger age groups. This might be due to the fact that women at this age group will be economically independent, in a stable marital relationship, and have completed their education unlike the younger age groups.

Clients having greater than 4 children [AOR = 4.28, (CI 1.24–14.71)] were 4 times more likely to commit induced abortion. This finding is in line with a study in Spain [24] in which those clients who have some children were more likely to have induced abortion than those who have no children. This may be true because women who had several pregnancies would have the tendency not to have additional children so they may tend to avoid unwanted pregnancies by inducing their current pregnancy.

Those patients who want the pregnancy [AOR = 0.44, (CI 0.14–0.65)] were less likely to have induced abortion. This finding is similar to another study conducted in Addis Ababa [19]. The immediate explanation for this is that a woman seeking induced abortion usually have unplanned or unwanted pregnancy which in turn is related to lack of wide spread information about family planning and its utilization.

Strength of this study is that it used primary data by directly interviewing the owners of the problem, whereas the limitation of the study is that since abortion is a sensitive issue and legally restricted in Ethiopia, patient interviews may introduce social desirability bias which may result in under reporting.

## 5. Conclusion

Generally, the study revealed a high level of induced abortion. Unwanted pregnancy is high among abortion patients in the study area. Educational level, age of the patient, number of pregnancy, and whether or not the pregnancy is wanted were factors significantly associated with induced abortion. Increased access to high-standard family planning service, post-abortion care, strict counseling about family planning method reminder, and partner involvement in family planning service should be emphasized.

## Figures and Tables

**Table 1:** Sociodemographic characteristics of postabortion patients in health institutions of Guraghe zone, South Ethiopia, from January to March, 2010.

Variables (*n* = 400)	Number	Percentage (%)
Age (mean 25.3 (SD ± 6.4))		
15–19	91	22.75
20–24	99	24.75
25–29	94	23.5
30–34	66	16.5
35+	50	12.5
Marital status		
Married	227	56.8
Single	160	40
Divorced	9	2.3
Widowed	4	1
Occupational status		
House wife	185	46.3
Student	134	33.5
Daily laborer	29	7.3
Office worker	21	5.3
Teacher	9	2.3
Commercial sex worker	8	2
House maid	7	1.8
Others	7	1.8
Educational status		
Illiterate	130	32.5
Read and write	46	11.5
Primary education	99	24.8
Secondary education	87	21.8
Tertiary education	38	9.5
Ethnic group		
Guraghe	319	79.8
Amhara	46	11.5
Oromo	23	5.8
Others	12	3
Religion		
Orthodox	166	41.5
Muslim	182	45.5
Protestant	39	9.8
Catholic	13	3.3

**Table 2:** Reproductive history of postabortion clients in health institutions of Guraghe zone, South Ethiopia, from January to March, 2010.

Variables (*n* = 400)	Frequency	Percentage
Number of pregnancies**		
<2	207	51.8
3–5	128	32
6–8	61	15.2
9 and above	4	1
Delivery		
None	185	46.3
1	40	10
2–4	130	32.5
5–7	43	11.25
8 and above	2	0.5
Previous abortion		
Yes	68	17
No	332	83
Frequency of previous abortion (68)		
Once	59	86.8
Twice	8	11.7
Three times	1	1.5
Current pregnancy wanted		
Yes	98	24.5
No	302	75.5
Current pregnancy induced/interfered		
Yes	49	12.3
No	351	87.7

**Current pregnancy was included.

**Table 3:** Reasons for unwanted pregnancy, place and methods of interference to the current pregnancy among post abortion clients in health institutions of Guraghe zone, South Ethiopia, from January to March, 2010.

Variables	Number	Percent
Reason for unwanted pregnancy (302)		
Contraceptive failure	47	15.6
Forget to take contraceptives	102	33.8
Partner pressure	122	40.4
Do not know contraceptives	31	10.3
Place of interference to current pregnancy (*n* = 49)		
Health institutions	25	51
Patient's house	22	45
Inducers house	2	4
Method used for interference		
Plastic	15	30.6
Medication (oral, vaginal and injection)	16	32.7
Herbs	18	36.7
Doer of induced abortion		
Health workers	25	51
Traditional birth attendant	4	8.2
Self	20	40.8
Sex of who assisted patient		
Male	17	58.6
Female	10	34.5
Not reported	2	6.9

**Table 4:** Fertility awareness, pregnancy intentions, and family planning method provision among postabortion patients in health institution of Guraghe zone, South Ethiopia, from January to March, 2010.

Variables	Number	Percentage
Patient opinion on fertility return after abortion		
Within two weeks	98	24.5
One month	123	30.8
Three months	110	27.5
Six months	57	14.3
Above six months	10	2.5
No response	2	0.5
Future pregnancy plan		
Never	162	40.5
Within three months	70	17.5
Within two years	18	4.5
Above two years	131	33.1
No response	18	4.5
Got FP method		
Yes	226	56.5
No	174	43.5
Reasons for not getting FP		
No one raised the issue	47	11.8
Changed my mind	96	24.0
Referral	9	2.3
Other health reasons	3	0.8
Contraceptives were not available	19	4.8

NB: FP = family planning.

**Table 5:** Factors associated with induced abortion in health institutions of Guraghe zone, South Ethiopia from January to March 2010.

Variables	Induced abortion		COR (95% CI)	AOR (95% CI)
	Yes	No		
Age				
15–19	12	79	1	1
20–24	11	88	1.22 (0.51–2.91)	0.91 (0.36–2.26)
25–29	11	83	1.15 (0.48–2.75)	0.63 (0.20–1.97)
30–34	11	55	0.75 (0.53–5.73)	0.15 (0.04–0.55)*
30+	4	46	1.75 (0.53–5.73)	0.31 (0.06–1.54)
Marital status				
Married	25	202	1	1
Single	24	149	0.77 (0.42–1.40)	1.351 (0.58–3.14)
Education				
Illiterate	10	120	1	1
Read and write	9	37	0.34 (0.13–0.91)*	0.35 (0.12–1.01)
Primary (1–8th)	16	83	0.43 (0.19–1.00)*	0.26 (0.13–0.88)*
Secondary and above	14	109	0.44 (0.18–1.04)	0.28 (0.08–1.01)
Occupation				
Employed	2	35	1	1
Unemployed	47	316	0.38 (0.09–1.65)	1.33 (0.11–15.51)
Religion				
Christians	44	268	1	1
Muslims	5	83	1.07 (0.58–1.95)	1.18 (0.60–2.34)
Current pregnancy				
Unwanted	42	390	1	1
Wanted	7	61	2.10 (0.91–4.84)	0.44 (0.14–0.65)*
Previous abortion				
No	42	260	1	1
Yes	7	91	1.26 (0.54–2.94)	1.17 (0.39–3.48)
Number of pregnancy				
Less than 4	44	268	1	1
Greater than 4	5	83	2.73 (1.05–7.10)*	4.28 (1.24–14.71)*

*Significant at 95% Confidence level.
